# Molecular mechanisms, genetic mapping, and genome editing for insect pest resistance in field crops

**DOI:** 10.1007/s00122-022-04060-9

**Published:** 2022-03-10

**Authors:** Shabir H. Wani, Mukesh Choudhary, Rutwik Barmukh, Pravin K. Bagaria, Kajal Samantara, Ali Razzaq, Jagdish Jaba, Malick Niango Ba, Rajeev K. Varshney

**Affiliations:** 1 Mountain Research Center for Field Crops, Sher-e-Kashmir University of Agricultural Sciences and Technology of Kashmir, Khudwani, J&K 192101 India; 2 ICAR-Indian Institute of Maize Research (ICAR-IIMR), PAU Campus, Ludhiana, Punjab 141001 India; 3 Center of Excellence in Genomics and Systems Biology (CEGSB), International Crops Research Institute for the Semi-Arid Tropics (ICRISAT), Hyderabad, 502324 India; 4 Department of Genetics and Plant Breeding, Centurion University of Technology and Management, Paralakhemundi, Odisha 761211 India; 5 Centre of Agricultural Biochemistry and Biotechnology, University of Agriculture Faisalabad, Faisalabad, 38040 Pakistan; 6 Intergated Crop Management, International Crops Research Institute for the Semi-Arid Tropics (ICRISAT), Hyderabad, 502324 India; 7 International Crops Research Institute for the Semi-Arid Tropics (ICRISAT), BP 12404, Niamey, Niger; 8 State Agricultural Biotechnology Centre, Centre for Crop and Food Innovation, Food Futures Institute, Murdoch University, Murdoch, WA 6150 Australia

## Abstract

**Key message:**

Improving crop resistance against insect pests is crucial for ensuring future food security. Integrating genomics with modern breeding methods holds enormous potential in dissecting the genetic architecture of this complex trait and accelerating crop improvement.

**Abstract:**

Insect resistance in crops has been a major research objective in several crop improvement programs. However, the use of conventional breeding methods to develop high-yielding cultivars with sustainable and durable insect pest resistance has been largely unsuccessful. The use of molecular markers for identification and deployment of insect resistance quantitative trait loci (QTLs) can fastrack traditional breeding methods. Till date, several QTLs for insect pest resistance have been identified in field-grown crops, and a few of them have been cloned by positional cloning approaches. Genome editing technologies, such as CRISPR/Cas9, are paving the way to tailor insect pest resistance loci for designing crops for the future. Here, we provide an overview of diverse defense mechanisms exerted by plants in response to insect pest attack, and review recent advances in genomics research and genetic improvements for insect pest resistance in major field crops. Finally, we discuss the scope for genomic breeding strategies to develop more durable insect pest resistant crops.

## Introduction

Insect damage is one of the major biotic constraints limiting the productivity and production of major field-grown crops. Besides feeding on several plant parts, insects also act as carriers or vectors for various plant parasitic viruses (Chang et al. 2021) and cause extreme plant damage. Despite extensive efforts of plant breeders, over US $70 billion are invested annually world-wide for the management of insect pest damage (Bradshaw et al. 2016). Heavy reliance on chemical pesticides may not be feasible as they provide temporary benefits, often with adverse environmental hazzards and in some instances can worsen farmer’s overall pest problems (Akhtar et al. 2009). One alternative to chemical control of insect pests is host plant resistance (HPR). Although the potential of HPR has not been fully explored, it is environmental friendly and compatible with other control means. The major challenge today is to develop insect pest resistant varieties that can increase and sustain crop productivity, in a rapidly changing world.

Crop wild relatives (CWRs) and/or non-domesticated species possess several desirable genes that can confer resistance to insects (Mammadov et al. 2018; Khan et al. 2020). However, introgression of valuable insect resistance genes into an elite cultivar preferrable for commercial release is usually a time-consuming and laborious task. Specifically, if a wild relative or non-domesticated crop species is the donor genetic material, the process of gene introgression can take more than a decade (Plaisted et al. 1992). A focus on genomics-assited breeding holds potential to address many of these challenges and the data to support this is beginning to arise in several crop species (Varshney et al. 2021a). Genomics-assisted breeding technologies are increasingly being utilized to enhance the yield, resistance to biotic and abiotic stresses, and quality traits of crops. For instance, dissection of QTLs can help to identify and develop molecular markers specific for target traits. By utilizing such markers, marker-assisted selection strategy provides a platform for deployment of identified QTLs to develop insect pest resistant crops (Varshney et al. 2014). Technological advances in such modern breeding techniques facilitate the use of wild species, landraces, and traditional varieties as sources of desirable genes for crop improvement (Pandey et al. 2016; Bohra et al. 2021). Introgression of these resources in cultivated gene pool will ultimately help in increasing the genetic diversity of cultivated crop germplasm together with providing agronomically beneficial traits (Varshney et al. 2019). Complementary to breeding strategies, targeted genome editing facilitated by clustered regularly interspaced short palindromic repeats (CRISPR)/CRISPR-associated nuclease protein (Cas) systems has enabled precise, efficient, and targeted manipulation of target genes associated with insect resistance and agronomically important traits (Gui et al. 2020; Wang et al. 2018a).

In this review, we highlight recent advances in mapping and genome editing of insect pest resistance loci in major crops. We first describe diverse resistance mechanisms employed by plants against insect pest attack. We then provide an overview of recent advances in the identification and map-based cloning of insect resistance QTLs in major cereal and pulse crops. Further, we describe the use of CRISPR/Cas9-mediated genome editing in overcoming certain long-standing issues in breeding for insect resistance. We conclude by highlighting some new research areas that are now becoming active, and which can be explored for enhancing insect pest resistance.

## Plant resistance mechanisms against insect pests

**Fig. 1:**
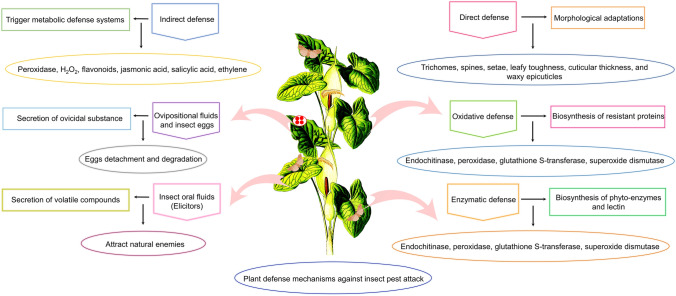
Diverse defense mechanisms employed by plants for insect pest management. The figure illustrates different defense mechanisms imparted by plants, including various direct and indirect defenses, which help them to counteract insect herbivory

One of the important factors influencing crop productivity in controlled and natural vegetation is the arms race between plant and insect pests. To counteract insect herbivory, a wide array of defenses are imparted by crop plants to decrease the risk of impairment and reduction in productive capacity (Mitchell et al. 2016). Plant defense mechanisms include various direct and indirect approaches to defend themselves against insect attack. Direct defense mechanisms include specialized morphological structures produced by plants, while indirect mechanisms include secondary metabolism activated in plants. Few examples of defense mechanisms observed in field-grown crops in response to insect pest attack are provided in Fig. 1.

### Morphological barriers

The plant morphological structures significantly contribute toward host plant resistance in response to pest herbivory. The defensive leaf structures of the plant safeguards itself by the development of dense trichomes, spines, setae, as well as leafy toughness, cuticular thickness, and release of waxy epicuticles (Peterson et al. 2016). In particular, morphological traits such as leaf glossiness, plant vigor, and leaf-sheath pigmentation are responsible for imparting resistance against Sorghum shoot fly, *Atherigona soccata*, in sorghum (Mohammed et al. 2016; Arora et al. 2021). In sweet corn, husk tightness showed significant resistance against corn ear worm, *Helicoverpa zea* (Cameron and Anderson 1966; Wiseman and Davis 1990). Trichomes adversely impact the ovipositional sites and feeding behavior of insect pests and block their mobility to leaf epidermis by obstructing their movement over plant surface (Sánchez and Morquecho-Contreras 2017). Trichomes on cowpea pods were also found to confer resistance to the pod sucking bug, *Clavigralla tomentosicollis* Stål (Hemiptera, Coreidae) (Boukar et al. 2020). In some grasses, trichomes tend to hinder sap-feeding or leaf-chewing insects to a greater extent (Hartley et al. 2015).

### Plant secondary metabolites

The host plant species induces a response upon insect attack to decline the survival of the insect and its reproductive rate (War et al. 2012). There are two major pathways through which plants recognize insect attack, viz*.* oral secretions (OS) of insects and ovi-positional fluids (Karban and Baldwin 1997). The OS of insects is composed of certain compounds which are recognized by the host plant cells. These compounds are known as elicitors or herbivore-associated molecular patterns, which trigger plant defense mechanism upon insect attack. A specific class of elicitors in plants called herbivore-associated elicitors (HAEs) are responsive toward folivorous insects (Bonaventure et al. 2011). During insect folivery, the HAEs can act in a diversified manner ranging from different structures to varied enzymes and in modified forms of lipids [e.g. fatty acid–amino acid conjugates] (Mattiacci et al. 1995; Eichenseer et al. 1999). For example, a compound named volicitin from the oral secretions of beet armyworm caterpillars induced maize seedlings to emit volatile compounds which can attract several parasitic wasps and other natural enemies of caterpillars. Shinya et al. (2016) demonstrated that the entire action of *Mythimna loreyi* OS, is significantly contributed by high molecular mass elicitor(s) fraction. The accumulation of reactive oxygen species (ROS) and phytoalexins were detected in the cells of rice, which were strongly induced by the high molecular mass elicitor(s) containing fraction. The HAEs can also act as modified forms of sulfur-containing fatty acids (caeliferins). For instance, the 16-carbon analog of caeliferin is responsible for induction of volatile organic compounds (VOCs) in maize upon herbivory of grasshopper species (*Schistocerca Americana*) that decoys the regular enemies of herbivores (Alborn et al. 2007). An apterous *Aphis craccivora* was attracted to cowpea leaves pre-infected with low population density (~ 10 individuals) of both alate and apterous aphid forms, but they were repelled if plants were pre-infected with higher population density groups (> 50 individuals) of aphids. This represents a natural adaptation to colonize fresh leaves or those with the least population of conspecifics, to avoid intraspecific competition (Jaba et al. 2010).

The persistent contact of insect eggs and their ovipositional fluids on plant surface can induce defense responses. Plants are very sensitive to ovipositional fluids which elicit defense responses upon its attachment to plant surface. For instance, in rice, hypersensitive responses were noticed after the attachment of lepidopteran, hemipteran, and coleopteran insect eggs, which led to desiccation and detachment of the eggs from the plant surface (Shinya et al. 2016). The presence of predator (ladybird beetle, *Cheilomenes sexmaculata*) eggs on aphid-infested cowpea failed to attract its own species of other ladybird beetles. This might be due to the presence of marker pheromone/spacing pheromone-like chemicals, which are released from the egg mass deposited in aphid colonies (Jaba et al. 2013). Furthermore, plants can also get rid of insect eggs through secretion of ovicidal substances. Salerno et al. (2013) showed that maize leaves elicit defense by retaining egg parasitoids upon the secretion of accessory reproductive glands of *Sesamianon agrioides*. Yang et al. (2014) reported a natural defense mechanism which induced watery lesions and high egg mortality against the whitebacked planthopper (*Sogatella furcifera*) in rice.

The major role of flavonoids in plants lies in providing defense against abiotic or biotic stresses. For instance, a plant strengthener named 4-FPA (4-fluorophenoxyacetic acid) regulates peroxidases, H_2_O_2_, and flavonoid production that directly cause flavonoid polymer formation. The rise in phenolic polymer accumulation in rice parenchyma cells was found to be closely related to a declining ability of the whitebacked planthopper (WBPH) *Sogatella furcifera* to reach the plant phloem (Wang et al. 2020). Notably, pigeonpea genotypes (ICPH 3461, ICPH 3762, BSMR 853, ICPL 332 WR, ICPH 2740, and ENT 11) with better pod wall thickness, high non-glandular trichome density, and high phenol, tannins, and flavonoids content showed improved tolerance to pod borer complex (Ambidi et al. 2021). In a recent study, the resistance of cowpea to aphids, *Aphis craccivora* (Koch), was found to be linked to their low sucrose levels and high levels of kaempferol and quercetin (aglycones of phenolic compounds) (Togola et al. 2020).

### Phytohormones and herbivore-induced plant volatiles

To confront challenges of biotic stress from herbivores, plants have refined their defense strategies. Jasmonic acid (JA), salicylic acid, and ethylene as phytohormones contribute towards inducing indirect plant defense mechanisms (Thaler et al. 2012; Zhang et al. 2013). The lipid-derived phytohormone, jasmonate, proved to be very important in inducing defense responses toward the insect. For instance, quick jasmonate synthesis was consistently elicited upon insect herbivory which was then sensed by the F-box protein COI1 to promote recruitment of Jasmonate Zim Domain repressors for ubiquitination and degradation purpose. These activities stimulated the transcriptional factor release and thereby triggered defense against insect attack (Wang et al. 2019). The JA content in plants draws the attention of several parasitoids and predators on insects. According to Ye et al. (2019), rice leaves emit the indole volatile upon fall armyworm (*Spodoptera frugiperda*) caterpillar attack. As a result, *OsMPK3* gets primed by indole activity that leads to transcription of *OsWRKY70* and other jasmonate biosynthesis genes. This overall activity results in further assembly of bioactive oxylipins viz*.*, JA precursor OPDA (12-oxophytodienoic acid) and JA and consequently declines larval growth, weight gain and damage. Salicylic acid elicitation has obvious implications toward plants, pathogens, insect pests, and their natural enemies. The release of volatiles related to salicylic acid defenses can draw attention of natural enemies both above- and below-ground, and can assist in reducing insect pest populations on crop (Filgueiras et al. 2019). In maize, application of foliar methyl salicylate attracted the subterranean entomopathogenic nematode *Heterorhabditis amazonensis*, which recruited herbivore-induced cues, infected insect larvae feeding on plant roots and enhanced the biological control of corn, which is fed by adult *Diabrotica speciosa,* the corn rootworm pest (Filgueiras et al. 2016).

Specified mixtures of herbivore-induced plant volatiles (HIPVs) (such as phenylpropanoids, terpenoids, sulphur containing compounds, nitrogen containing compounds, fatty acid-derived compounds, and isothiocyanates) are emitted by plants upon insect herbivores’ attack (Aartsma et al. 2017; Dicke and Lucas-Barbosa 2020). HIPVs can promote defense priming by stimulating quick response in intact tissues of plants (Erb et al. 2015; Mauch-Mani et al. 2017). Further, HIPVs can also attract parasitoids and predators that can contribute toward controlling the attacking insect (Aartsma et al. 2017; Xiu et al. 2019; Mbaluto et al. 2020). According to Aljbory and Chen (2018), about 24 species of predators and 34 species of parasitoids were attracted to volatiles emitted from plants infested by herbivory insect pests. The egg parasitoid *Cotesia sesamiae* (Cameron) (Hymenoptera: Braconidae) is attracted to volatiles emitted by maize plants on which *Chilo partelllus* (Swinhoe) (Lepidoptera: Crambidae) lays its eggs. The genetic basis for *C. partelllus* induced indirect defense attracting *C. sesamiae* was investigated in maize genotypes (Tamiru et al. 2020). A genome-wide association study revealed 101 single nucleotide polymorphisms (SNPs) strongly associated with the trait. Within a 10 Mb region of the genome next to these SNPs, 33 candidate genes were found that may code for the trait, of which 7 were terpene synthase genes (*tps2*, *tps3*, *tps4*, *tps5*, *tps7*, *tps9,* and *tps10*) (Tamiru et al. 2020).

### Defensive proteins

The requirement of nutrition for insect is quite comparable to other animals, and any disparity in the absorption and usage of plant proteins by insect herbivores causes harsh impact on insect physiology. The biotic stress resulting from insect attack stimulates gene alteration, which consequently alters the proteins both qualitatively and quantitatively. These changes significantly contribute in signal transduction as well as oxidative defense. The infestation by spotted stem borer (*Chilo partellus*) was found to induce more protein suppression but selective defensive protein aggregation in *Sorghum bicolor*. These proteins were mainly concerned with stress and defense, small molecule biosynthesis, amino acid metabolism, catalytic and translation regulation activities (Tamhane et al. 2021). The vegetative insecticidal protein (VIP), e.g., VIP1 and VIP2, present in the supernatant of vegetative *Bacillus cereus* culture, have been demonstrated to confer toxic effects on insects (Gupta et al. 2021). The VIP3 isolated from *B. thuringiensis* supernatant, which is similar to Cry proteins in terms of toxicity potential, possess insecticidal activity against Lepidopteran pests. These proteins stimulate gut paralysis, and then total lysis of gut epithelium cells, leading to larval death (Gupta et al. 2021). The endochitinases, peroxidases, and glutathione *S*-transferase produce over catalytic activities of these defensive proteins (Alseekh et al. 2020). Likewise, another protein called superoxide dismutase was found at a high concentration in wheat, and showed strong coliniearity with high resistance against invading aphids (Lightfoot et al. 2017).

Plant protease inhibitors are small proteins that naturally occur in plants (especially leguminous plants) and provide defense against a certain number of insect pests. They bind to trypsin in the insect gut that affects the synthesis and regulation of alimentary proteases. Ultimately, digestion and absorption of nutrients are disrupted leading to the death of the insect. For instance, protease inhibitors from cowpea (cowpea trypsin inhibitor) and soybean conferred resistance to a wide range of insect pests including lepidopterans (Zhao et al. 2019).

### Plant lectins

Plant lectins are proteins that promote carbohydrate ligand binding, which aid in obtaining the sensations from environmental signals and convert them into phenotypic responses. The down-stream signaling cascades are required for these processes to arise, usually mediated by interacting proteins. Together, all these processes play a significant role in plant resistance mechanism (Esch and Schaffrath 2017). In recent past, the use of proteins carrying jacalin-related lectins gained importance into the plant resistance research areas. In a recent study, transgenic tobacco plants expressing Hvt-lectin resulted in 100% mortality of *Helicoverpa armigera* and *Spodoptera litura* within up to 96 h after infestation (Rauf et al. 2019). A jacalin-related lectin, Orysata from rice species was found to be antagonistic against *Spodoptera exigua* and *Acyrthosiphon pisum* by lowering larval weight gain and obstructing development (Atalah et al. 2014). Hence, it is predicted that Orysata could be effectively used against biting-chewing and piercing-sucking insect pests.

### Defensive enzymes

The phyto-enzymes impair nutrient uptake of insects through electrophile formation and have become one of the key attributes of plant defense against insects. One of the most crucial enzymes is chitinase, which is also an integral component of insect integument. A soybean seed coat chitinase fraction incorporated in artificial cotyledons resulted in up to 90% mortality and up to 87% decrease in larval mass of the insect *Callosobruchus maculatus* (Silva et al. 2018). Lipoxygenase is the first enzyme in the octadecanoic pathway which is a prominent link for the synthesis of the signaling molecule, which is associated in plant defense stimulation. The lipoxygenase-encoding gene was down-regulated in a susceptible genotype, while the basal expression remained level in the wheat genotype showing resistance against *Rhopalosiphum padi* (Correa et al. 2020). The accumulation of green leaf volatiles aided in declining the aphid preference, and the action of non-glandular trichomes as a physical barrier enabled uninterrupted lipoxygenase-encoding gene expression (Correa et al. 2020).

## Insect resistance QTLs identified in major field crops

### Rice

Brown planthopper (BPH) is one of the most destructive pests that reduces rice production in Asia. Qiu et al. (2014) carried out genetic mapping for brown planthopper resistance in 93–11/T12 F_2_ population and located *Bph7* gene on the long arm of chromosome 12, between SSR markers RM28295 and RM313. *Bph7* is responsible for 38.3% phenotypic variation of BPH resistance in the F_2_ population. The gene mapping of *Bph7* can be utilized for map-based cloning and eventually in development of BPH-resistant lines in rice (Jaganathan et al. 2020). Similarly, a F_2:3_ population developed from a cross between BPH susceptible (Zhenshan 97) and BPH resistant (IR65482-17) genotype was utilized for mapping three QTLs for seedling resistance and feeding rate to BPH. Among the identified QTLs, *qBph4.2* on chromosome 4 (between SSR markers, RM261 and SNP S1) exhibited the largest effect by contributing phenotypic variation of about 36–44% (Hu et al. 2015a). Further, Wu et al. (2014) mapped *Bph28(t)* between markers Indel55 and Indel66 in two different F_2_ mapping populations developed by crossing the common resistant parent, DV85 with susceptible japonica variety Kinmaze and Indica 9311. Fine mapping of such genes using advanced genomics technologies (Jaganathan et al. 2020) will greatly contribute toward marker-assisted gene pyramiding programs for insect resistance. Recently, Yuexiong et al. (2020) and Yang et al. (2020) mapped *Bph35* and *Bph38*, respectively, on chromosome 4 in rice. These studies indicated the importance of chromosome 4 in rice, which consists of a large number of QTLs mapped for BPH resistance (Table 1). QTL mapping studies using a RIL population led to the identification of three QTLs, namely *qSBPH2*, *qSBPH3*, and *qSBPH7.1* located on chromosomes 2, 3, and 7, respectively, for small brown planthopper (SBPH) resistance that exhibited a cumulative phenotypic variation of 35.1% (Wang et al. 2013). Van Mai et al. (2015) used two independent F_2_ populations derived from a cross of ASD7 × Taichung 65 and mapped two QTLs (*qBPH6* and *qBPH12*) for BPH resistance and one QTL (*qGRH5*) for green rice leafhopper (GRH) resistance. Recently, Thein et al. (2019) identified four major QTLs, namely *qGRH2, qGRH4, qGRH5*, and *qGRH11* in African wild rice *O. longistaminata* accession W1413 derived backcross populations, with *qGRH2* being a novel QTL for GRH.

**Table 1:** A list of key QTLs mapped/ fine mapped/ cloned for insect pest resistance in some field crops

Crop	QTL	Mapping population	Cross	Marker type	Environment	Chromosome/linkage group	Stress	References
Rice	*Bph38*	F_2:3_	9311 × RBPH327	SSR (*RM16563* and *RM16763*); *YM112* and *YM190*	Greenhouse	4	Brown planthopper (BPH) and whitebacked planthopper (WBPH)	Yang et al. (2020)
Rice	*Bph35*	F_2:3_	9311 × RBPH660	InDel (*PSM16* and *R4M13*)	Greenhouse	4	BPH	Yuexiong et al. (2020)
Rice	*Bph36* and *Bph27*	F_2:3_, BC_1_F_2_	KW × RBPH16 and HHZ × RBPH17	InDels (S13 and X48), *RM16766* and *RM17033*	Greenhouse	4S and 4L	BPH	Li et al. (2019)
Rice	*Bph38*(t)	BC_1_F_5_	Khazar × Huang–Huan–Zhan	SNP (*693*, *369*)	Greenhouse	1L	BPH	Balachiranjeevi et al. (2019)
Rice	*bph39(t)* and *bph40(t)*	BC_1_F_2_	[RPBio4918-230S line- (IRGC81848 × Swarna)] × Swarna	–	Greenhouse and field	–	BPH biotype 4	Akanksha et al. (2019)
Rice	*Bph37*	F_2:3_	KWQZ × IR64	*RM302* (SSR) and *YM35* (InDel)	Greenhouse	1	BPH biotype 2	Yang et al. (2019)
Rice	*Bph34*	F_2_, F_2:3_	PR122 × IRGC104646	SNP (*AX-95952039* and *AX-95921548*), SSR (*RM16994* and *RM17007*)	Field and screenhouse	4L	BPH	Kumar et al. (2018)
Rice	*Bph30*	BC_1_F_2_, BC_2_F_2_, NILs	AC-1613 × 9311	*SSR-28* and *SSR-69*, *RM16278* and *RM16425*, *RM16294* and *RM16299*	Field	4S	BPH	Wang et al. (2018b)
Rice	*Bph33*	NILs, F_2:3_	Kolayal × 9311 and Poliyal × 9311	InDels (*H25* and *D17*)	Greenhouse	4S	BPH	Hu et al. (2018)
Rice	Two QTLs (*qBph4.3* and *qBph4.4*)	F_8_ RILs	TN1 × Salkathi	SSR (*RM551* and *RM335*, *RM335* and *RM5633*)	Greenhouse	4S	BPH biotype 4	Mohanty et al. (2017)
Rice	10 QTLs	F_2:3_	MR276 × RathuHeenati	*RM231*, *RM588* and *RM204*	Greenhouse	1, 3, 6, 7, 9, 10, 12	BPH biotype 3	Shabanimofrad et al. (2017)
Rice	*Bph31*	F_2_	Jaya × CR2711–76	InDels (*PA26* and *RM2334*)	Glasshouse	3L	BPH biotype 4	Prahalada et al. (2017)
Rice	*Bph32*	F_14_	Ptb33/163B//163B	SSR (*RM19291* and *RM8072*)	Greenhouse	6S	BPH	Ren et al. (2016)
Rice	*qBPH6* and *qBPH12*	F_2_	ASD7 × Taichung 65	SSR-*RM8120* and *RM8200* (*qBPH6*), *RM3326* and *S20103* (*qBPH12)*	NM	6S and 12L	BPH	Van Mai et al. (2015)
Rice	*QBph3* and *QBph4*	BC	IR02W101 × Zhenshan 97	SSR-*RM514* and *J412* (*QBph3*), *RM261* and *RM307* (*QBph4*)	Greenhouse	3L and 4S	BPH	Hu et al. (2015b)
Rice	*QBph4.2*	F_2:3_ and BC	IR65482-17 × Zhenshan 97	Co-segregate with Indel *XC4-27* (between SSRs *RM261* and *SNP S1*)	Greenhouse	4S	BPH	Hu et al. (2015a)
Rice	*bph29*	NILs	TR539 × TN1	InDels (*BYL8* and *BID2*)	Greenhouse	6S	BPH	Wang et al. (2015)
Rice	*Bph26/bph2*	NILs	ADR52 × Taichung 65	SNP (*DS-72B4* and *DS-173B*)	NM	12L	BPH	Tamura et al. (2014)
Rice	*bph7*	F_2_ and BC	93–11 × T12	SSR (*RM3448* and *RM313*)	Greenhouse	12L	BPH	Qiu et al. (2014)
Rice	*Bph28(t)*	F_2_	DV85 × Kinmaze (*japonica*) and DV85 × 93–11 (*indica*)	InDels (*InDel 55* and *InDel 66*)	Greenhouse	11	BPH	Wu et al. (2014)
Rice	*Bph3*	BC and F_2_	RathuHeenati × 02428	SSR (*RH078*, *W4*, *RM8213*, *RM16533* and *RM5953*)	Greenhouse	4	BPH	Liu et al. (2015)
Rice	*Bph27*	BC	Teqing × GX2183	SSR (*RM16846* and *RM16888*)	Greenhouse	4L	BPH	Huang et al. (2013)
Rice	*Bph27(t)*	F_2:3_	Balamawee × *japonica* cv. 02428	InDels (*Q52* and *Q20*)	Greenhouse	4L	BPH	He et al. (2013)
Rice	*Bph25* and *Bph26*	F_2_ and NILs	ADR52 × Taichung 65	SSR (*S00310* and *RM5479*)	NM	6S and 12L	BPH	Myint et al. (2012)
Rice	*Bph6*	F_2_ and NILs	Swarnalata × 93–11	STS (*Y19* and *Y9*)	Greenhouse	4 L	BPH	Qiu et al. (2010)
Rice	*bph4*		TN1 × Babawee, Babawee × KDML105	SSR (*RM589* and *RM586*)	NM	6S	BPH	Jairin et al. (2010)
Rice	*qSBPH7.1*	RILs	N22 × USSR5	SSR (*RM234* and *RM429*)	Greenhouse	2,3 and 7	Small BPH	Wang et al. (2013)
Rice	*qSBPH12-a1*	BC and F_2_	02428 × RathuHeenati	SSR (*RM519* and *RM3331*)	Greenhouse	12L	Small BPH	Tuyen et al. (2012)
Rice	*Qsbph3b,Qsbph11d* and *Qsbph11e*	BC	Nipponbare × Kasalath	SSR (*C80–C1677*, *R1506–C950* and *S2260-G257*)	Greenhouse	3 and 11	Small BPH	Duan et al. (2010)
Rice	*qWDS-6* and *qWNS-12*	F_2:3_	TN1 × SinnaSivappu	SSR (*RM589-RM539* and *SSR12-17.2 RM28487*)	Greenhouse	6 and 12	WBPH	Ramesh et al. (2014)
Rice	Four QTLs (*qGRH2*, *qGRH4*, *qGRH5*, *qGRH11*)	Five BC_3_F_3_ and seven BC_3_F_4_	*O. sativa* ssp. *japonica* cv. ‘Nipponbare’ × African wild rice *O. longistaminata* accession W1413	SSR	Greenhouse	2L, 4S, 5S and 11L	Green rice leafhopper (GRH)	Thein et al. (2019)
Rice	*qGRH5*	F_2_	ASD7 × Taichung 65	SSR (*RM6082* and *RM3318*)	NM	4S	GRH	Van Mai et al. (2015)
Rice	*gm12*	F_2:3_	KDML105 × MN62M	SNP markers (*S2_76222* and *S2_419160*)	Greenhouse	2S	Asian rice gall midge	Leelagud et al. (2020)
Rice	*gm3*	RILs	TN1 × RP2068-18-3-5	SSR (RM17480 and *gm3SSR4*)	Greenhouse	4L	Gall midge	Sama et al. (2014)
Rice	*Gm8*	RILs	TN1 × Aganni	SSR (*RM22685* and *RM22709*)	Greenhouse	8	Gall midge	Sama et al. (2012)
Rice	*Gm11t*	RILs	TN1 × CR57-MR1523	SSR (*RM28574* and *RM28706*)	Greenhouse	12	Gall midge	Himabindu et al. (2010)
Rice	*Gm10*	F_2:3_	BG 380–2 × Susceptible cv	–	Natural condition	–	Asian rice gall midge	Kumar et al. (2005)
Rice	*Gm 9*	F_2:3_	Madhuri Line 9 × MW10	–	Natural condition	–	Asian rice gall midge	Shrivastava et al. (2003)
Rice	*qAfrGM4*	BC	ITA306 × BW348-1, ITA306 × TOG7106 and ITA306 × TOS14519	SNP using KASP assay	Greenhouse	4	African rice gall midge	Yao et al. (2016)
Wheat	*H35* and *H36*	RILs	SD06165 × OK05312	SNP (*SDOKSNP7679*), (*SDOKSNP1618- SDOKSNP8089*)	Greenhouse	3BS and 7AS	Hessian fly	Zhao et al. (2020)
Wheat	*h4*	RILs	Bobwhite × Java and Overley × Java	KASP (*KASP3299* and *KASP1871*)	Greenhouse	1AS	Hessian fly	Niu et al. (2020)
Wheat	*H7* and *H8*	F_5:6_ and F_4:5_	Bobwhite × Seneca	*KASP6A205* and *KASP6A215*	Greenhouse	6AL and 2B	Hessian fly	Liu et al. (2020)
Wheat	*H34*	RILs	Ning7840 × Clark	SSR and SNP (*Xsnp921*-*Xsnp274)*	Greenhouse	6B	Hessian fly	Li et al. (2013)
Wheat	*QHf.osu-1A*^*d*^ and *QHf.osu-1A*^*74*^	DH	Duster × Billings	SSR—*Xcfd15* and PCR based-*OPRA1*, Genotyping-by-sequencing (*GBS07851* and *GBS10205*)	Greenhouse	1A	Hessian fly	Li et al. (2013)
Wheat	*QHf.osu-1A*, *QHf.osu-2A*	RILs	Jagger × 2174	SSR (*Xcfa2153*)	Greenhouse	1A, 2A	Hessian fly	Tan et al. (2013)
Wheat	*QHf.uga-6AL (HR61)* and*QHf.uga-3DL*	RILs	26R61 × AGS 2000	SSR (*Xgwm427-wPt731936* and *Xcfd4b-Xgwm52*, respectively)	Green House	6AL and 3DL	Hessian fly	Hao et al. (2013)
Wheat	*QDn.unlp genes*	DH	Spark × Rialto	SSR (Xpsp3103, *Xgdm3* and *Xpsp3094*)	Greenhouse	4DS, 5DS and 7AL	Russian wheat aphid	Ricciardi et al. (2011)
Wheat	*Ei1*	DH	Cham6 × IG139883, Cham6 × IG139431	SNP iSelect assay and Candidate gene-based KASP markers (*IWB66138* and *BS00022785*)	Artificial screen cages	4BS	Sunn Pest	Emebiri et al. (2016)
Maize	62 QTNs	Associationmapping panel	341 tropical maize lines	DArTseq markers	Field	All 10	Maize weevil and fall armyworm	Badji et al. (2020)
Maize	15 QTLs	RILs	Population 84 × Kilima	SSR (*bnlg1909-umc1884*, *Phi094-umc2189*)	Field	1,2, 3, 4, 8, and 10	Maize weevil	Castro-Álvarez et al. (2015)
Maize	*Aphid resistance QTL*	RILs	B73 and Mo17	*AC213878* and *AC204415*	Greenhouse	4	Corn leaf aphid	Betsiashvili et al. (2015)
Maize	4 Major QTLs for root damage, root regrowth, and root size traits	IRIL–IBM Recombinant Inbred Line	IBM – Intermated B73 × Mo17	SSR and SNP (*umc1395, umc1321; bnlg1598, umc1123; csu3, mmp61; bnlg1867, mmp13*)	Field	1 and 6	Western corn rootworm	Brkić et al. (2020)
Maize	4 and 3 putative QTLs for RDR, RRG and RSZ	DHLs	(NGSDCRW1 × AG1 and LH51 × CRW8-1) top crossed with PHZ51	SNP	Field	7, 9, 10 and 6, 8, respectively	Western corn rootworm	Bohn et al. (2018)
Maize	*c3 NI (q03.165)*	F_2_, BC, and DH	FS8B × B86, UR2 × Mo47	SNP (*MAGI_14202* and *MAGI_72398*)	Artificial cages	3	Western corn rootworm	Hessel (2014)
Maize	Six QTLs (three for tunnel length, one each for kernel resistance, stalk damage, and yield)	RIL	A637 × A509	SNP	Field	5, 8, 9, and 10	Mediterranean corn borer	Jiménez-Galindo et al. (2017)
Maize	8 QTLs for resistance traits like tunnel length, stalk damage, stalk lodging, kernel resistance, and grain yield	RILs	B73 × CML103	SNP (*30460922–73132746, 30460922–73132746, 9498146–88522572*)	Artificial	1, 5 and 6	Mediterranean corn borer	Samayoa et al. (2015)
Maize	*HDMBOAGlc QTL*	RILs	B73 × CML322	*PZA03189.4* and *PMH5098.25*	Greenhouse	1	Corn leaf aphid	Meihls et al. (2013)
Soybean	*Raso2*	RILs	Williams 82 × PI 366121	SNP using Golden Gate assay (*BARC-042815–08424* and *BARC-015945–02020*)	Plant growth chambers	7	Foxglove aphid	Lee et al. (2015)
Soybean	*Raso1*	BC	Toyomusume × Shokukei-10	SSR (*Gm03-11* and *Gm03-12*)	Plant growth chambers	3	Foxglove aphid	Ohnishi et al. (2012)
Soybean	*Rag6* and *Rag3c*	F_3:4_	E08934 × E00003	*MSUSNP08-2* and *Satt209*; *MSUSNP16-10* and *Sat_370*	Field and greenhouse	8 and 16	Aphid	Zhang et al. (2017)
Soybean	*R_P746*	F_2:3_	P746 × Dongnong 47	SSR (*Satt335* and *BARCSOYSSR_13_1508*)	Greenhouse	13	Aphid	Xiao et al. (2014)
Soybean	*QTL_13_1* and *QTL_13_2*	RILs	Wyandot × PI 567324	Oligo Pool Assay containing SNPs	Greenhouse and field environment	13	Aphid	Jun et al. (2013)
Soybean	*rag3* and* rag1b*	F_4:5_	IA2070 × E06902	SSR and SNP (*Gm16_6262227_C_T*–*Gm16_6424067_A_G* and *Satt435-BARCSOYSSR_07_0295*)	Field and greenhouse	16 and 7	Aphid	Bales et al. (2013)
Soybean	*Rag1*	BC_4_F_2_	Dowling × Dwight	SNP markers (*46169.7* and *21A*)	Plant growth chambers	7	Aphid	Kim et al. (2010a)
Soybean	*Rag 2*	F_2:3_	LD02-4485 × (Ina × PI 200538	SNP (*KS9-3* and *KS5*)	Green House	13	Aphid	Kim et al. (2010b)
Soybean	*Rag 3*	F_4_ derived lines	PI 567543C × E00003	SSR (*Sat_339* and *Satt414*)	Greenhouse and field environment	16	Foxglove aphid	Zhang et al. (2010)
Soybean	*qRWF-1* and *qRWF-5-1*	F_2_	Huapidou × Qihuang26	SSR (*satt071-satt147* and *satt619-satt545*)	Field environment	1 and 5	Whitefly	Zhang et al. (2013)
Chickpea	Nine main-effect QTLs for *H. armigera* resistance component traits	RILs	ICC 4958 × PI 489777	Axiom^®^*CicerSNP* Array	Field environment, and controlled conditions	CaLG01, CaLG03, CaLG04, and CaLG07	*Helicoverpa armigera*	Barmukh et al. (2020)
Cowpea	*Thr-1*, *Thr-2*, and *Thr-3*	RILs	IT93K503-1 × CB46	AFLP (*ACC-CAT7, ACG-CTC5*, and *AAG-CAT1*)	Field environment	5 (*Thr-1, Thr-2)* and 7 (*Thr-3*)	Thrips	Muchero et al. (2010)
Cowpea	*QAc-vu7.1*	RILs	CB27 × IT97K-556–6	SNP (*1_0912–1_0391*)	Field environment	7	Aphid	Huynh et al. (2015)
Mungbean	Two QTLs for bruchid resistance and one QTL for pod sucking bug	F_2_, F_2_	Sunhwa × Jangan, Sunhwa × TC1966	SSR (*MB87* and *COPU11*), and *COPU06*	Controlled conditions -growth chambers	–	Bruchid and Pod sucking bug	Hong et al. (2015)
Rice bean	*Cmpd1.5* and *Cmpd1.6*	F_2_, F_2_	LRB238 × LRB26, JP100304 × LRB26	SRAP markers (*E2M9-270 and E12M7311*), and SRAP marker and *SSR-CEDG259*, respectively	Controlled conditions-growth chambers	4 and 9	Bruchid	Venkataramana et al. (2016)
Pea	4 QTLs (*BpSI.I, BpSI.II*, *BpSI.III* and *BpLD.I*)	F_8:9_	*P. sativum* ssp. *syriacum*accession P665 × *P. sativum* ssp.* sativum*cv. Messire	DArTseq markers	Field environments	LGI, LGII, LGIV and LGIV, respectively	Pea weevil	Aznar-Fernández et al. (2020)
Pea	2 QTLs	F_7:8_	*P. sativum* ssp.* syriacum* accession P665 × *P. sativum* ssp. *sativum* cv. Messire; *P. fulvum* accessions, P660 × P651	DArTseq markers	Field environments	–	Pea weevil, aphid, and rust	Barilli et al. (2019)
Pea	*COR2, COR4b,* and *SCR2*	F_2_	*P. sativumcv. Pennant* × *P. fulvum (ATC113)*	SSRs (*AA179-AA189*), (*AB28-AA297*), (*AA179-AA189*)	Glasshouse	2 and 4	Pea weevil	Aryamanesh et al. (2014)

Rice gall midge (RGM) is another important pest of rice affecting its production globally. Sama et al. (2014) carried out the mapping of QTLs for gall midge resistance in RILs developed from a cross between TN1 (susceptible) × RP2068-18-3-5 (resistant) using SSR markers. A significant association was observed between gene and phenotype based on flanking markers, *RM17480* and *gm3SSR4*. Based on the sequence polymorphism, ‘*gm3del3*’ was cloned and efficiently utilized as a functional marker for introgressing *gm3* gene in the elite bacterial blight resistant cultivar Improved Samba Mahsuri (B95-1), via marker-assisted selection (MAS) approach. Leelagud et al. (2020) mapped *gm12* on chromosome 2 in the F_2:3_ population derived from the cross of KDML105 and MN62M, wherein SNP markers (S2_76222 and S2_419160) were found to flank *gm12*. This gene can be extensively used for the identification of RGM biotypes in Thailand and Southeast Asia. African rice gall midge (AfRGM) has proved to be a very destructive pest in the areas of irrigated and lowland African ecologies. Three independent bi-parental rice populations (ITA306 × BW348-1, ITA306 × TOG7106, and ITA306 × TOS14519) were developed for the identification of QTLs resistant to AfRGM, followed by meta QTL (mQTL) analysis studies to identify the conserved genomic regions across different genetic backgrounds. Out of the total 28 QTLs identified, a major QTL *qAfrGM4* was mapped on chromosome 4 in ITA306 × TOS14519 population, and this QTL exhibited 34.1% phenotypic variation. Meta-analysis revealed that most of the mQTLs were background specific, except one minor effect mQTL (chromosome 1) that was common in the TOS14519 and TOG7106 genetic backgrounds. This is the first reported QTL for AfRGM resistance and further fine mapping is under process for its efficient utilization in MAS (Yao et al. 2016).

### Wheat

Sunn Pest (*Eurygaster integriceps*) is a pest of serious concern in wheat. Mapping studies with 90 k SNP iSelect assay and candidate gene-based KASP markers in two separate DH populations derived from Cham6 × IG139431 and Cham6 × IG139883, respectively, led to the identification of a major QTL for resistance to sunn pest, *Ei1* on chromosome 4BS (Table 1). The *Ei1* was mapped on chromosome 4B between markers, IWB66138 and BS00022785, and was found to be very close to other agronomically important genes like GA-insensitive dwarfing gene, Rht-B1 (Emebiri et al. 2016). Hessian fly (HF), *Mayetiola destruc*tor is a destructive pest of wheat globally. Gene pyramiding is the best approach to achieve resistance to HF owing to the availability of multiple biotypes that are virulent to different wheat HF resistance genes, and this approach relies upon the identification of linked DNA-based markers. Li et al. (2013) developed a RIL population by crossing Ning7840 with the HF resistant genotype Clark for identification of QTLs governing HF resistance. A major QTL designated *H34* for resistance to fly biotype *GP* exhibiting 37.2% phenotypic variation was mapped on chromosome 6B, between markers Xsnp921 and Xsnp2745*.* Further, a major QTL for Hessian fly resistance, *QHf.osu-1A,* was mapped on chromosome 1A using SSRs in hexaploid wheat RILs derived from a cross between Jagger and 2174. This was followed by the identification of two new loci for HF resistance namely *QHf.uga-3DL* and *QHf.uga-1AL* (HR61) on chromosome 3 and 6, respectively, in RILs derived from a cross of 26R61 with AGS 2000 (Hao et al. 2013). Recently, Zhao et al. (2020) mapped a major QTL, *H35* on chromosome 3BS in SD06165 × OK05312 RIL population. Niu et al. (2020) mapped a recessive gene, *h4* on chromosome 1AS in RILs derived from two separate crosses (Bobwhite × Java and Overley × Java) using KASP markers.

### Maize

Maize leaf aphid (*Rhopalosiphum maidis*) is one of the most destructive pests affecting maize production globally. A RIL mapping population developed by crossing B73 and CML322 was used to map QTLs for leaf aphid resistance. This study led to the identification of *HDMBOAGlc* on the chromosome 1 between markers, PZA03189.4 and PMH5098.25 (Meihls et al. 2013). Castro-Álvarez et al. (2015) carried out QTL mapping for maize weevil resistance in RILs derived from a cross between population 84 and Kilima. A total of 15 QTLs for maize weevil résistance mapped on 6 different chromosomes exhibited a range of phenotypic variation between 14% and 51%. These QTLs hold potential to be utilized for tropical maize improvement through MAS. Mediterranean corn borer (MCB), *Sesamianon agrioides*, is a major pest of maize, in Mediterranean countries. Jiménez-Galindo et al. (2017) mapped six QTLs (three for tunnel length, and one each for kernel resistance, stalk damage, and yield) for MCB resistance on chromosomes 5, 8, 9, and 10 in A637 × A509 based RILs. A double haploid (DH) population developed by crossing UR2 with Mo47 was used to map QTLs for resistance to western corn rootworm. Among a total of 21 QTLs identified, a major QTL *c3 NI (q03.165)* was mapped on chromosome 3 between SNPs, MAGI_14202 and MAGI_72398. It was also found that a herbivore stress response governing *sps2* gene lied within the identified QTL interval (Hessel 2014). Recently, Brkić et al. (2020) mapped four major QTLs for root damage, root regrowth, and root size traits on chromosome 1 and 6 using maize IBM Intermated RILs (B73 × Mo17 based). These QTLs were found to co-locate with genomic regions governing plant defense against herbivory.

### Sorghum

Sorghum shoot fly, *Atherigona soccata* (Rondani) is one of the most damaging pest affecting world-wide sorghum production. A total of four QTLs were identified, and SBI-05 was found to contain the major QTL for non-preference to oviposition; while SBI-01, SBI-07, and SBI-10 contributed to shoot fly resistance (Kiranmayee et al. 2015). Furthermore, assessment of phenotypes led to the identification of two resistant lines for each QTL region present on chromosomes SBI-01, SBI-07, and SBI-10 in ICSB 29004 × Parbhani Moti (Gorthy et al. 2017). In another study, a joint analysis for *Busseola fusca* and *C. partellus* revealed that marker *CS132-2* was co-localized for leaf toughness and stem tunneling traits on two individual QTLs identified; thus, suggesting that the two traits can be improved using the same linked marker (Muturi et al. 2021).

### Soybean

The soybean aphid (*Aphis glycines* Matsumura) is an important pest of soybean [*Glycine max* (L.) Merr.]. Jun et al. (2013) mapped two major QTLs, namely *QTL_13_1* and *QTL_13_2*, for aphid resistance on chromosome 13 using RILs developed from a cross between Wyandot and PI 567324. The study revealed that *QTL_13_1* and *QTL_13_2* were mapped very close to already reported loci, *Rag2* and *Rag4*, respectively (Jun et al. 2013). Xiao et al. (2014) mapped *R_P746* between SSRs, *Satt334,* and *Satt335*, on chromosome 13 in a P746 × Dongnong 47 derived F_2:3_ mapping population comprising of 312 individuals. These linked markers will prove to be valuable in MAS-based aphid resistance breeding program in soybean. Further, Zhang et al. (2017) mapped two QTLs, *Rag6* and *Rag3c*, on chromosomes 8 and 16, respectively, and these QTLs were validated in two related populations with different genetic backgrounds. These QTLs were contributed by E08934, an advanced breeding line derived from the wild soybean *Glycine soja* 85-32; thereby indicating the importance of wild relatives in conferring tolerance to biotic stresses. Foxglove aphid, *Aulacorthum solani* (Kaltenbach), is a hemipteran insect of destructive nature in soybean. RILs derived from a cross between Williams 82 and PI 366121 were used for mapping of foxglove aphid resistance through antibiosis and antixenosis. Mapping was carried out with the help of Golden Gate assay-based SNP markers. This study resulted in the identification of a major QTL on chromosome 7 which was later named as *Raso2* to differentiate from the earlier reported QTL, *Raso1* (Lee et al. 2015).

### Chickpea

In chickpea, *Helicoverpa armigera* (Hübner) causes up to 100% yield losses in the tropical regions of the world (Patil et al. 2017). In a recent study, Barmukh et al. (2020) used an intraspecific RIL population (ICC 4958 × PI 489777) to map QTLs for *H. armigera* resistance component traits (Table 1). A total of nine main-effect QTLs and 955 epistatic QTLs explaining up to 42.49% phenotypic variation were mapped for multiple *H. armigera* resistance component traits. Interstingly, a QTL cluster on linkage group CaLG03 harboring main-effect QTLs for three component traits, was predicted to be of particular relevance for improving *H. armigera* resistance in elite chickpea cultivars (Barmukh et al. 2020).

### Cowpea

Thrips, *Megalurothrips sjostedti* Trybom and aphid, *Aphis craccivora* Koch are among the most damaging insect pests of cowpea in Africa (Kusi et al. 2019). SNP-based mapping of QTLs for thrips resistance identified major QTLs such as *Fthp28*, *Fthp87,* and *Fthp129* on chromosomes 2, 4, and 6 accounting for 24.5%, 12.2%, and 6.5% of the total phenotypic variation, respectively. Transgressive segregation was observed toward the susceptible phenotype. Both additive and non-additive effect QTLs were observed, with additive effects being predominant (Sobda et al. 2017). Furthermore, SNP-based mapping of QTLs for aphid resistance was carried out in a RIL population developed from a cross between California blackeye cultivar (CB27) and a resistant African breeding line (IT97K-556-6). This study resulted in the identification of a major QTL on chromosome 7, which was validated in a related F_2_ population (Huynh et al. 2015).

### Mungbean

Bruchid (*Callosobruchus chinenesis* L.) and pod sucking bug (*Riptortus clavatus* Thunberg) are pests of serious concern in mungbean during the reproductive stage and seed storage. Two QTLs for bruchid resistance and one QTL for pod sucking bug resistance were identified in two independent F_2_ populations derived from a cross of Sunhwa with Janganand, and Sunhwa with TC1966. The linked SSR markers hold promise to be successfully utilized for cloning of bruchid and bean bug resistant genes (Hong et al. 2015).

### Rice bean

Bruchids (*Callosobruchus maculatus*) is a major pest of stored seeds in rice bean (*Vigna umbellata*). Venkataramana et al. (2016) identified QTLs for damage caused to the seed related to bruchid resistance in two F_2_ populations derived from the cross of a common susceptible parent, LRB26 with LRB238 and JP100304. SSR and SRAP based genotyping resulted in the mapping of two major QTLs, *Cmpd1.5* and *Cmpd1.6* exhibiting 67.3 and 77.4% phenotypic variation, respectively.

### Pea

Pea weevil, *Bruchus pisorum*, is one of the most destructive pests decreasing the production of field pea (*Pisum sativum*) globally. QTL mapping for traits associated with pea weevil resistance, such as cotyledon and pod wall/seed coat resistance, was performed in an interspecific population derived from a cross between the cultivated field pea and *P. fulvum* (resistance source) (Aryamanesh et al. 2014). This study led to the mapping of three major QTLs on linkage groups LG2, LG4, and LG5 for cotyledon resistance, and two major QTLs for pod wall/seed coat resistance on LG2 and LG5 exhibiting upto 80 and 70% phenotypic variation, respectively. These identified QTL markers may prove to be crucial in the screening of pea germplasm for pea weevil resistance genes (Aryamanesh et al. 2014). Recently, Aznar-Fernández et al. (2020) used DArTseq markers to map four QTLs, *BpSI.I*, *BpSI.II, BpSI.III*, and *BpLD.I* for weevil seed infestation and larval development on linkage groups LGI, LGII, LGIV and LGIV, respectively.

## Breeding crops for insect pest resistance

Over the past decades, a large number of insect pest resistance QTLs have been identified in major field crops. However, only a limited number of actionable targets are known due to a lack of fine mapping and functional characterization. There is a rising need to clone and characterize the candidate genes underlying the identified QTLs, using fine mapping and map-based cloning approaches (Jaganathan et al. 2020). Such genes would shed light on the molecular mechanisms of insect resistance in crop plants.

The ultimate objective of mapping and cloning insect pest resistance genes, and unraveling the underlying defense mechanism is to facilitate the breeding of insect-resistant crop varieties, which represents an efficient, cost-effective, and environmental-friendly pest control strategy. Recent advances in genomics and sequencing technologies have opened new avenues for rapid identification of genetic variation underlying crop performance and have improved the efficiency of breeding. The significance of sequence variations in the ability of the plants to regulate specific traits has been further uncovered by digging deeper into the genomes (Varshney et al. 2021b). This has facilitated the initiation of the next level of biotechnological intervention, referred to ‘Genome editing’. This engrossing strategy enables modifications in the genome by adding, deleting, or editing specific DNA sequences, thereby presenting opportunities for use in plants, animals, and humans. In the current scenario of limited agricultural land and a higher load of insect pests on crop plants, genome editing holds enormous potential in combating insect pests and expediting crop improvement for future food security.

## Genome editing strategies for engineering insect pest resistance

In recent years, genome editing has emerged as the most promising technology to cope up with the challenges associated with agriculture production. This technology has provided unprecedented opportunities to develop improved crop genotypes having higher yield and better adaptability under increasing environmental fluctuations. Among several other plant genome editing technologies, the CRISPR/Cas system has emerged as one of the most widely used systems due to its cost-effectiveness, simplicity, and high efficiency (Zhu et al. 2020). CRISPR/Cas9 is continuously expanding its toolbox with new discoveries and innovations (Razzaq et al. 2019). Till date, several insect resistance QTLs have been identified in crops (Table 1), with each QTL having only a small effect on the phenotype while interacting with each other. Moreover, QTLs do not follow a simple Mendelian pattern of inheritance and are extremely difficult to study and manipulate. Genome editing holds promise in overcoming these limitations by offering tools to associate genetic polymorphisms with phenotypic variations. CRISPR-based QTL editing can be utilized to incorporate numerous preferred quantitative trait alleles directly into elite crop varieties, thereby preventing the need for intensive crossing (Shen et al. 2018). This strategy will be particularly suitable for editing QTLs that are present in low recombination regions of the genome. Importantly, the CRISPR multiplex technique can be used to manipulate a blend of candidate QTLs or all target genes present within the QTL region, resulting in alterations to measurable phenotypes.

**Fig. 2:**
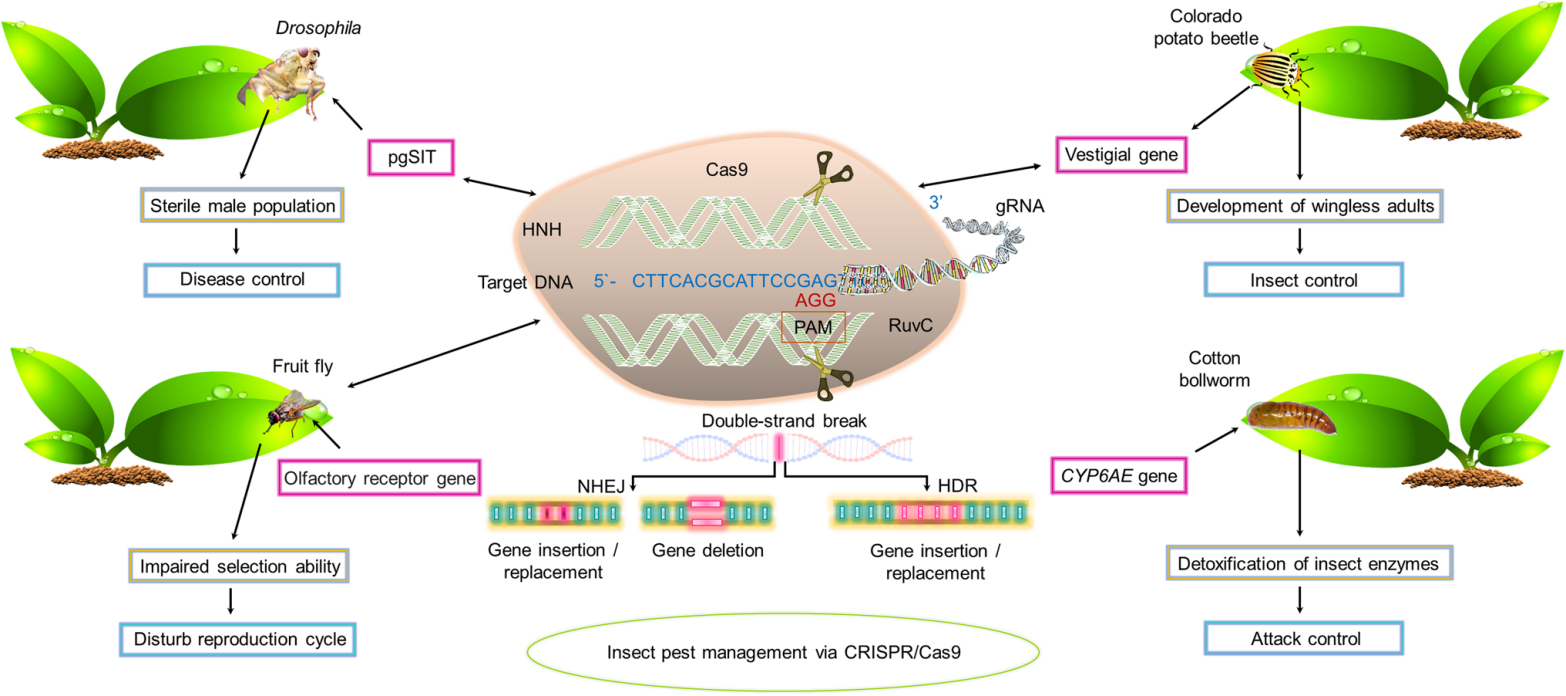
The CRISPR/Cas9-mediated genome editing applications for insect pest resistance in plants. The Cas9-gRNA complex targets desired seuquence in the DNA and produces a double stranded break (DSB) at the 3′ upstream PAM sequence. This results in gene insertion/deletion through non-homogous end joining (NHEJ) mechanism, while gene insertion via homology directed repair (HDR) process. Different genes and receptor genes can be knocked-out to control the insect pest population. *gRNA* Guide RNA, *NHEJ* Non-homogous end joining, *HDR* Homology directed repair, *DSB* double standard break, *pgSIT* precision guided sterile insect technique

An interesting aspect of managing insect pests via genome editing has the benefit of altering both plants and insects pests (Fig. 2). While recent advances in genome editing have transformed insects to make them less effective toward crop damage, plant genomes are being modified to make plants more effective in repelling insect pests. Here, we discuss some key research efforts being pursued and those which can be anticipated toward genome editing in crops for insect management.

### Genome editing in insects to alter and attenuate pest population

The utilization of CRISPR/Cas-based gene editing techniques to develop insect-resistant plants and also for targeting the insect pest/pathogens to reduce their destructive abilities has conversely been limited. CRISPR-enabled tools have increasingly advanced their ability and versatility to target multiple genes and to modify specific traits within the insect genome. The major focus on the design of sophisticated techniques for vector delivery includes binary vectors to assist in precise genome editing in insect pests. It provides an excellent platform for addressing several environmental problems and public health concerns in sustainable way (Gantz and Akbari 2018). An interesting feature of insect pest management via genome engineering has significant benefits of altering both plants and insect genomes. Innovative investigations are being performed to modify plant genomes for enhancing their capability to limit insect pests from feeding on plants and disarmed insects to avert their attack (Tyagi et al. 2020). Kandul et al. (2019) reported a precision guided sterile insect technique (pgSIT) based on CRISPR/Cas9 mediated genome editing in *Drosophila*. The sterile insect technique (SIT) is an ecologically secure and well-established approach to repress insect populations. Multiple pgSIT systems were engineered efficiently in *Drosophila* to constantly produce 100% sterile male population. This model population of pgSIT was competitive and offered great ability to suppress disease vectors and insect pests (Kandul et al. 2019). Another pest management strategy induced via CRISPR/Cas9 technology was demonstrated by Gui et al. (2020) to study the biology of Colorado potato beetle (CPB), *Leptinotarsa decemlineata*, an important pest of Solanaceae family, including potatoes. CRISPR/Cas9 induced mutagenesis of vestigial gene (*vest*) developed wingless adults of CPB with no elytron formed. This study provides an excellent way forward to control the CPB in environmentally safe manner (Gui et al. 2020).

Targeting the genes responsible for mating partner identification and chemical communication using genome editing technology is another strategy to control insect pests. These two properties are very crucial to establish insect–plant interaction, like the olfactory receptors in insects that help to sense the odorant of a mating partner and to develop host-plant interaction via chemical signaling. Koutroumpa et al. (2016) mutated *Or83b* gene using CRISPR/Cas9 system, which caused defect in olfactory receptors and disturbed the selection of host for laying eggs. Likewise, *Orco* (olfactory receptor coreceptor) gene was disrupted in *Spodoptera litura*, which resulted in impaired selection ability of host plant and distraction of insect from finding a mating partner (Koutroumpa et al. (2016). Insects produce unique enzymes that can be used to overcome the plant defense systems by releasing detoxification chemicals. Targeting these detoxification genes can increase the susceptibility of insects, especially in polyphagous species. A CRISPR/Cas9 based genome editing in *H. armigera* was conducted to knock-down *CYP6AE* gene cluster, which led to detoxification of these harmful chemicals (Wang et al. 2018a). Taken together, such techniques of insect pest management have great potential to deter the insect from crops and avoid yield loss.

### Genome editing in plants for insect pest management

Editing plants against multiple fungal, viral, and bacterial diseases has been successful in several agricultural crops (Vats et al. 2019). However, genome editing in plants for insect pest management has been comparatively less explored. Here, we discuss some key examples of targeting plant genes via CRISPR-based editing for insect management and highlight the possibilities of potential plant defenses that can be engineered for editing-based crop protection. In some instances, insect behavior, immunity, and even development depend on vital chemical substances (VOCs, secondary metabolites, etc.) produced and secreted by the plants. This has been successfully reported in rice by mutating the *CYP71A1* gene through CRISPR/Cas9 nucleases. This gene encodes tryptamine 5-hydroxylase that stimulates the production of serotonin from tryptamine, and plays a crucial role in stunted growth of plant hoppers. The mutant population showed increased resistance against striped stem borer (*Chilo suppressalis*) and brown planthopper (*Nilaparvata lugens*) in rice (Lu et al. 2018).

Many insect pests identify host plants through the plants’ volatile cues, morphological features, plant phenology, visual cues, odor and taste clues, and oviposition sites, among others (Larsson et al. 2004). An insect selects a particular plant for its oviposition site based on the availability of desired feed for its young ones. Plant VOCs contain a mixture of volatiles, among which only some are detected by insects as clues for selection of hosts and oviposition site. Recent study demonstrates that a particular volatile blend can be utilized as a kairomone-mediated lure to attract the predator *Nesidiocrois tenuis*, for the biological control of major tomato pest *Tuta absoluta* (Meyrick) and *Trialeurodes vaporariorum* (Westwood) (Ayelo et al. 2021). Modifications of plant volatile blends via genome editing can serve as an effective strategy for insect pest management. That said, utmost care needs to be taken to ensure that the manipulation of volatile blends do not cause deleterious impact on beneficial insect/natural enemy population.

Plant morphological features play an important role in the ability of insect pests to recognize and damage a particular host. For instance, modification in pigmentation of plants has been found to alter insect host preferences. This phenomenon was demonstrated in a study by Malone et al. (2009), in which upregulation of anthocyanin pigmentation produced red leaves in a transgenic tobacco plant. This alteration in leaf color acted as a deterrent for the pests, *H. armigera* and *S. litura*, thereby confirming the significance of leaf color on host recognition in insect pests. Taken together, engineering of specific metabolic pathways in plants resulting in a change in plant visual appearance can be used as a plausible approach for CRISPR/Cas9-based editing for management of insect pests.

The CWRs have a wide range of pest resistance traits but lack desired agronomic traits such as high yield, fruit/grain size, preferable plant structure, among others (Bohra et al. 2021). For example, the wild tomato *Solanum pimpinellifolium* has been reported to be resistant to a wide range of arthropod pests including spider mites (Rakha et al. 2017). Multiplex CRISPR/Cas9 editing of six different genes in *S. pimpinellifolium* resulted in high yielding tomato lines with additional resilience properties from wild tomato, in a single generation (Zsögön et al. 2018). Based on the characteristics and molecular processes underpinning the target organism, this technique can be meticulously applied to different CWRs. Notably, de novo domestication of CWRs can be a game-changing method for the development of crops with improved insect pest management features.

## Conclusion and future perspectives

Recent advances in molecular breeding of major crops such as rice, maize, and wheat, have resulted in better yield, good quality, and biotic and abiotic stress resistance. Major emphasis should now be given to develop improved crop varieties that hold the best genotypic combinations and also contain broad-spectrum resistance to both insect pests and diseases. Importantly, the role of microbiomes (for instance, bacteria, fungi, endophytes, floral microbes, etc.) on plant resistance to insect pests can also be explored. On-going developments in plant genetics and biotechnology provide exciting possibilities for the control of pest populations in an environment-friendly manner. Stacking of multiple genes has increased the protection power against multiple harmful organisms, and has the added advantage of durability and reduced risk of emergence of new herbivore resistance. Insect resistance to transgenic crops can be delayed using strategies such as refuge crops, and high toxin expression, among others.

The use of modern genomic breeding technologies offer enormous potential to design insect resistant crops for the future. For instance, adoption of genomic selection, and haplotype-based breeding strategies will facilitate the assembly of superior haplotype combinations of insect resistance alleles in elite cultivars, thereby enabling informed decision-making in breeding programs (Bohra et al. 2020). Genetically modified crops are also being adopted in several countries but special attention should be given to food safety and resistance management. Local systems, their constraints and socio-economic implications should be strictly considered before the adoption of such genetically modified materials. Importantly, there is a growing need to pursue management strategies that reflect the pest biology, plant–insect interactions, and their effect on the natural enemies to prolong the usefulness of the resistant crops.

## Data Availability

Not applicable.
